# Strategic Information Processing from Behavioural Data in Iterated Games

**DOI:** 10.3390/e20010027

**Published:** 2018-01-04

**Authors:** Michael S. Harré

**Affiliations:** Complex Systems Research Group, Faculty of Engineering and IT, The University of Sydney, Sydney 2006, Australia; michael.harre@sydney.edu.au; Tel.: +61-2-9351-8642

**Keywords:** behavioural economics, game theory, synergy, transfer entropy, matching pennies, cognitive strategies

## Abstract

Iterated games are an important framework of economic theory and application, at least since the original work of Axelrod’s computational tournaments of the early 80’s. Recent theoretical results have shown that games (the economic context) and game theory (the decision-making process) are both formally equivalent to computational logic gates. Here these results are extended to behavioural data obtained from an experiment in which rhesus monkeys sequentially played thousands of the “matching pennies” game, an empirical example similar to Axelrod’s tournaments in which algorithms played against one another. The results show that the monkeys exhibit a rich variety of behaviours, both between and within subjects when playing opponents of varying complexity. Despite earlier suggestions, there is no clear evidence that the win-stay, lose-switch strategy is used, however there is evidence of non-linear strategy-based interactions between the predictors of future choices. It is also shown that there is consistent evidence across protocols and across individuals that the monkeys extract non-markovian information, i.e., information from more than just the most recent state of the game. This work shows that the use of information theory in game theory can test important hypotheses that would otherwise be more difficult to extract using traditional statistical methods.

## 1. Introduction

An evolution in economic thinking has recently been transforming the field as researchers reconsider the foundations on which economics is based. An example of this is Mirowski [1] who has argued for seeing economic markets as evolving computational entities. In this view the focus of research is on the computational laws of the market, not the laws of human nature, see [2] for the history of this idea and further reading. Tesfatsion [3] has countered that Agent Based Models (ABMs) [4], in which the individual economic actor and her computational ability to make bounded rational decisions, should be the focus of computational economic modelling. This article extends recent work [5] in which both the agent and the economic structure are seen as computational processes, specifically as classical logic gates such as an XOR or an AND gate, and applied information theory to the task of understanding an agent’s computational strategy in an economic game. This work aims to illustrate the relationship between economic structure (i.e., the underlying game) and the decision making process (i.e., the strategies) of the agent as distinct but interrelated computations.

Game theory is one of the key methodologies through which we have come to understand the decision-making processes of economic agents when joint actions decide an individual’s reward. Initial research extended the static equilibrium models of von Neumann and Morgenstern [6] and Nash [7] in the mid-20th century. More recently, work has moved to developing a dynamic approach to economic interactions that has lead to game theory being applied to evolution [8], crowd dynamics [9], and ecological dynamics of large populations [10] amongst others. Dynamic games have also been studied by Axelrod [11] and Nowak [12] where the focus is on the time sequence of discrete decisions and the effectiveness of the strategies that these sequences encode. One of the early key results to come from this work is that the Tit-for-Tat (TfT) strategy is often the most effective strategy to play in the Prisoner’s Dilemma game, even against cognitively sophisticated opponents [11]. A follow up study [13] showed that the Win-Stay, Lose-Switch (WSLS) strategy could be more effective in the Prisoner’s Dilemma, and it was recently suggested [14] and then formally shown [5] that these strategies are indistinguishable for the Matching Pennies game but not for the Prisoner’s Dilemma.

The economics of individual interactions studied in game theory can be separated into two distinct aspects: games provide a taxonomy of the strategic interactions between agents, whereas game theory is a mathematical description of the choices made by agents with specific knowledge and cognitive abilities in the context of a specific game [15] (Chapter 1). Recently [5] this distinction was used to separate two distinct computational processes in games: the deterministic logic of the game structure and the computational process of a given behavioural strategy, where both games and decisions were mapped to computational logic and analysed using information theory. Moreover, this logico-cognitive framework can be readily extended to cellular automata, formal systems, and Turing machines (see [16] for a recent review), leading directly to universal computation and the undecidability of the dynamics of economics [17,18].

This article extends the theoretical analysis in [5] to the behavioural study of three rhesus monkeys playing iterated economic games (matching pennies) for rewards [19]. This study provides a very large and rich set of data in which the relationship between previous rewards, previous actions and the monkeys’ memories can be explicitly measured using information theory to provide a non-parametric estimate of the learned strategies of the monkeys in a competitive environment. While this dataset has been extensively studied in the past [14,19,20], the behavioural dynamics and information theoretical analysis provides an important complementary analysis to previous parametric statistical analysis.

## 2. Materials and Methods

### 2.1. Game Theory: Matching Pennies

Here we will consider normal form, two person, non-cooperative games in which the agents i=1,2 select between one of two possible acts (pure strategies): $a^{i}\in A^{i}$ (where we also define: $A=A^{1}\times A^{2}$). The joint acts of the two agents determine the utility for each agent *i*, $u^{i}:A\to I\;R$. We use $u^{i}$ to denote agent *i*’s utility function, taking joint action $a=a^{1}\times a^{2}$ as an argument, and $u_{n}$ as the utility value (a real number) for agent *i* in the *n*-th round of an iterated game. The monkeys that play these games are given a constant reward and this is arbitrarily set to 1 for success and 0 for failure, see the experimental protocol described in Section 2.3. The actions available to the agents and their subsequent utility values are given by the conventional bi-matrix notation for the matching pennies game (By convention *i* indexes the agent being considered, −i indexes the other agent):



agent −i

LeftRightagent *i*  Left  (1,0)(0,1)Right  (0,1)(1,0)

The intuition in this game is that there is a natural tension between the strategies the two players need to adopt in order to be able to maximise their reward: agent *i* wants both players to play either (Left, Left) or (Right, Right), whereas agent −i wants both players to play either (Left, Right) or (Right, Left). There is only one Nash equilibrium to the game (when played only once between the two players), in which both players uniformly at random choose either Left or Right. However, if the game is repeatedly played between the two agents Nowak has shown that strategies with a temporal component to them, such as TfT or WSLS, can have a better utility payoff than the Nash equilibrium [11] and learning effects might also be expected to play a role.

### 2.2. Probabilities and Information Theory

Information theory allows us to study the stochastic relationship between elements in a given system in order to see how they are variously correlated in a non-parametric fashion. In particular, by conditioning on different variables with different time delays we can infer important relationships between stochastic variables in order to see how their relationships are changing over time. In the Matching Pennies game, we have a number of stochastic variables we wish to track, specifically $a_{n}^{m}\in$ {left,right}, $a_{n}^{c}\in$ {left,right} and $u_{n}\in \left\{0,1\right\}$ are respectively the action (choice) of a monkey at time *n*, the action of the computer (see the experimental description) at time *n* and the payoff the monkey receives for these joint actions at time *n*. The total number of experiments (times the monkeys playing the game) is defined to be *N*; this changes for the different experimental set-up as well as for each monkey. The marginal probabilities over these variables are given by: $p_{n}\left(a^{m}\right)$, $p_{n}\left(a^{c}\right)$, and $p_{n}\left(u_{n}\right)$ where the time index n∈{1,…,N−500} are the empirical probabilities estimated over a sliding temporal window 500 interactions wide where the window slides up one time step at a time, i.e., the first window is from experiments 1 to 500 for which we calculate $p_{1}\left(a^{m}\right)$, the second is from experiments 2 to 501 for which we calculate $p_{2}\left(a^{m}\right)$, and so on. We also make use of the combined stochastic variable $S_{n}=\left\{a_{n}^{m},a_{n}^{c},u_{n}\right\}$. The joint and conditional probabilities such as $p_{n}\left(a_{n}^{m},u_{n-1}|a_{n-1}^{c}\right)$ represent the joint probability of a monkey’s action and the utility it received in the immediate past of that action, conditioned upon the action taken by the computer in the immediate past. The methods and information theory set-up that follow are identical with the Matching Pennies set-up of the previous study [5] and are reiterated here with only minor modifications for completeness.

The starting point of information theory is the Entropy:

(1) $$\begin{array}{ccc} H(x) & = & -\sum_{x^{i}\in X}p\left(x^{i}\right)\log_{2}p\left(x^{i}\right), \end{array}$$

measured in bits, and maximised if *x* is uniformly distributed over all $x^{i}\in X$ and zero if $p(x^{i})=1$ for any *i*. We will also use the mutual information and the conditional mutual information [21]:

(2) $$\begin{array}{ccc} I(x:y) & = & \sum_{x,y}p\left(x,y\right)\log\left(\frac{p(x|y)}{p(x)}\right) \end{array}$$

(3) $$\begin{array}{ccc} I(x:y|z) & = & \sum_{x,y,z}p\left(x,y,z\right)\log\left(\frac{p(x|y,z)}{p(x|z)}\right) \end{array}$$

The Transfer Entropy (TE) is also an important special case of the conditional mutual information [22,23], see [24] for a recent review. For two time series of stochastic variables *X* and *Y* with realisations $x_{n}$ and $y_{n}$ at time n∈{1,2,…}, the TE of $\left\{\left\{x_{n},y_{n}\right\},\left\{x_{n+1},y_{n+1}\right\},\ldots \right\}$ is the mutual information between one variable’s current state and the second variable’s next state conditional on the second variable’s current state:

(4) TY→X=I(yn:xn+1|xn)01234567890123456789012

(5) 012345678=∑xn,yn,xn+1p(xn,yn,xn+1)logp(xn+1|yn,xn)p(xn+1|xn).

We interpret this as the amount of information shared between *X* and *Y* once the history of *X* is conditioned upon.

In iterated games we wish to know how much information is shared between the state space $S_{n}$ and the next choice $a_{n+1}^{i}$. We assume that each agent *i* has a strategy $Z^{i}\left(\ldots \right)$ which the agent uses to take previous states of the system $S_{n}$ and generates an action at time n+1, this may depend on system states an arbitrary length of time l+1 into the past:

(6) $$\begin{array}{ccc} Z^{i}\left(S_{n}^{i},S_{n-1}^{i},\ldots ,S_{n-l}^{i}\right) & = & a_{n+1}^{i}, \end{array}$$

where $\left\{S_{n}^{i},S_{n-1}^{i},\ldots ,S_{n-l}^{i}\right\}$ is all of the information an agent uses in order to make a decision. A very simple 1-step $Z^{i}\left(S_{n}^{i}\right)$ is Tit-for-Tat (TfT) in which an agent *i* simply copies the previous act of the other agent [12,25]:

(7) $$\begin{array}{ccc} a_{n+1}^{i}\;\;=\;\;Z_{0}^{i}\left(a_{n}^{-i}\right) & = & a_{n}^{-i}. \end{array}$$

An *l*-step Markovian strategy is one in which the agent uses information from the previous *l* steps and an *l*-step Markovian game is a sequence of repeatedly played games where each agent has an *l*-step Markovian strategy.

### 2.3. The Experimental Set-up

For the details of the monkey protocol and the monkey-computer interface see the original article [19] and discussions in subsequent articles [14,20]. There is a complete statistical analysis of this data set, including neurological recordings, available in the original article. Here we briefly describe the computer algorithms that the monkeys were playing against. Each of the three monkeys reported here played the matching pennies game against three different computer algorithms, each game was played many thousands of times using each of the algorithms. Algorithm 1, monkey 1:35, 504 times; Algorithm 1, monkey 2:50, 143 times; Algorithm 1, monkey 3:70, 111 times; Algorithm 2, monkey 1:38, 557 times; Algorithm 2, monkey 2:28, 344 times; and Algorithm 2, monkey 3:45, 770 times. For the 0 algorithm (for which the results are not reported here) the computer chose each of the two targets randomly with equal probabilities and independently of all past decisions and outcomes of the game, corresponding to the Nash equilibrium in the matching pennies game.

In Algorithm 1, the computer sought to exploit systematic biases in the animal’s choices in order to minimise the monkey’s reward rate. All monkey choices, computer choices, and subsequent rewards are recorded in a given session, and this information is used to predict the animal’s next choice by testing a set of hypotheses. In the first hypothesis, the conditional probabilities of choosing each target given choices from the preceding *n* trials (n=0 to 4) were estimated. For the next hypothesis, each of the conditional probabilities were tested for the animal having chosen both targets with equal probability. If none of these are rejected, the computer selects either target with 50% probability. If a hypothesis is not rejected, the computer biases its next choice according to the probability with the largest deviation from 0.5 that was statistically significant. So in trial *n* of a session the conditional probability of a monkey making choice $a_{n}^{m}\in$ {Left, Right} based on the past history of moves was tested, there were five of these conditional probabilities, the simplest being: $p(a_{n}^{m})$ and the most extensive looked at the past four moves: $p(a_{n}^{m}|a_{n-4}^{m},a_{n-3}^{m},a_{n-2}^{m},a_{n-1}^{m})$.

In Algorithm 2, the computer considered both the rewards and the previous choices of the monkey in order to exploit any systematic bias. In addition to the hypotheses tested in Algorithm 1, Algorithm 2 tested the hypothesis that the monkey’s decisions were independent of prior choices and their payoffs in the preceding *n* trials (n=1 to 4). In this case we have a joint variable $\left\{u_{n},a_{n}^{m}\right\}\equiv ua_{n}\in \left\{1,0\right\}\times$ {Left, Right} and the statistical tests tested the conditional probability: $p(a_{n}^{m}|ua_{n-4},ua_{n-3},ua_{n-2},ua_{n-1})$. So in order to maximise the total reward a monkey needs to choose both targets with equal frequency and to make choices independently from previous choices and payoffs.

## 3. Results

All data analysis was carried out using the JIDT information theory toolbox for Matlab, see [26] for documentation and implementation.

### 3.1. 1-Step Monkey Memory and Total Information

**Figure 1:**
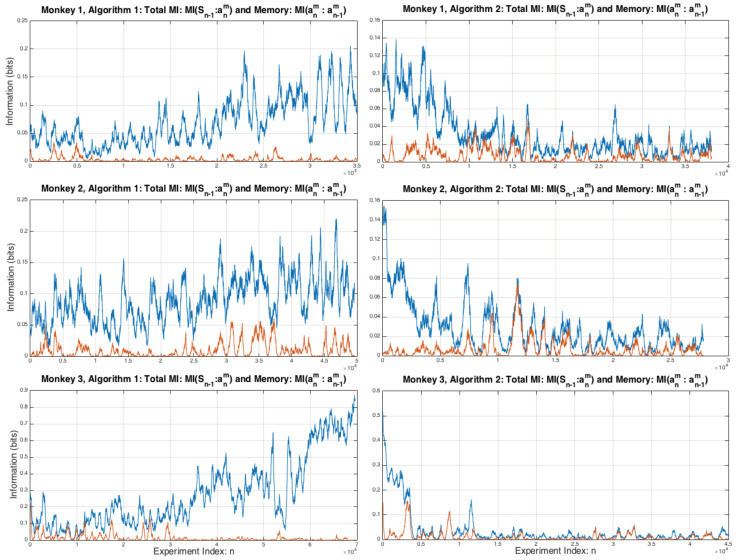
Total 1-step information from $S_{n}$ used by each monkey to make its next choice $a_{n+1}^{m}$ and the memory a monkey uses.

Figure 1 shows the mutual information between the joint variable $S_{n}$ and a monkeys next act $a_{n+1}^{m}$ measured by the mutual information $I\left(\left\{a_{n}^{m},u_{n},a_{n}^{c}\right\}:a_{n+1}^{m}\right)=I\left(S_{n}:a_{n+1}^{m}\right)$ and the amount of information the monkey uses from its last choice, i.e., its active memory: $I(a_{n}^{m}:a_{n+1}^{m})$. It can be seen that for Algorithm 1 all three monkeys gradually increase the total amount of information from the previous choice that is being used to make their next choice. In contrast, for Algorithm 2 the amount off information decreases over time as the monkeys use less and less of past information to make their next choice. The amount of active memory of past choices the monkeys are using makes up very little of the total information.

### 3.2. Pairwise and Higher Order Interactions

**Figure 2:**
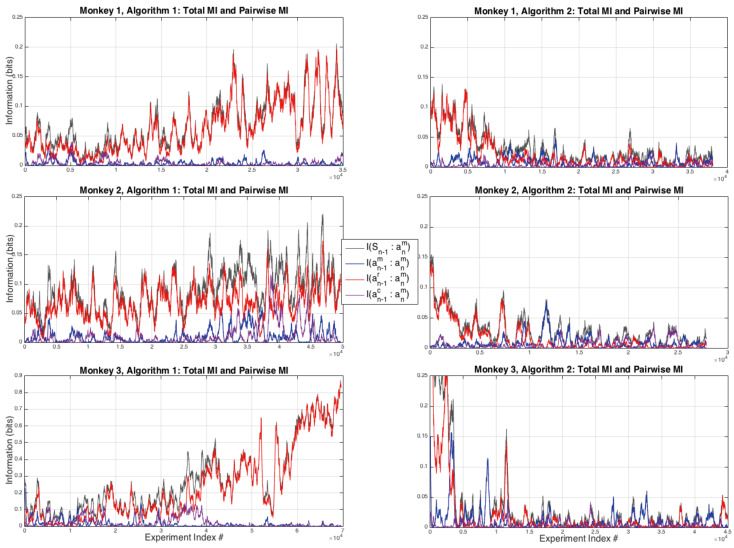
All pairwise interactions and total information. It can be seen that the largest pairwise contribution to the total information, for all three monkeys during Algorithm 1, is from the reward to the next choice the monkeys make. This contribution is significantly reduced for Algorithm 2. Note that there is only sporadic evidence of a pairwise interaction between the monkeys’ next choice and the computer’s previous choice: $I(a_{n}^{c}:a_{n+1}^{m})$ (purple curve).

**Figure 3:**
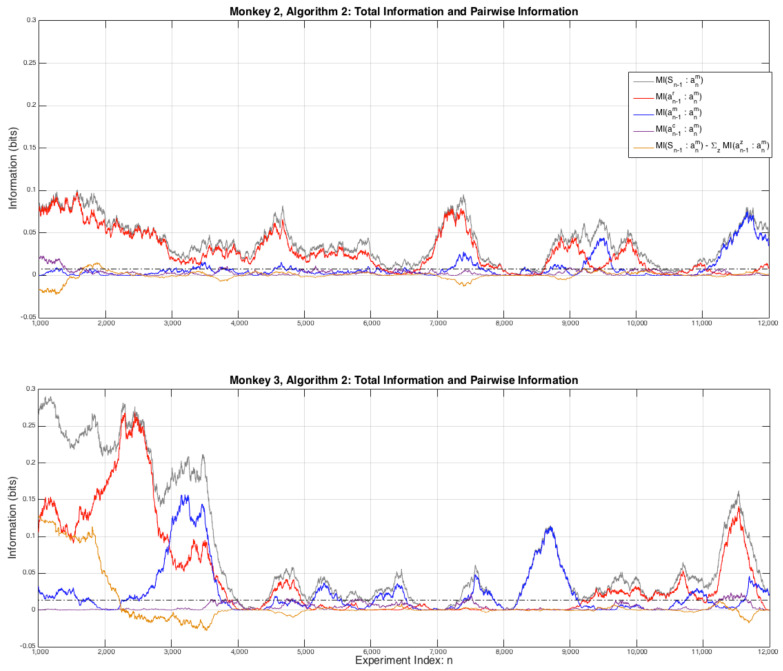
An expanded view of the first 12,000 games from Figure 2. For comparison the grey dashed line is the average information of unrelated data, calculated using exactly the same vectors as the results except that one vector is time shifted by n=3, here we have used: $I(a_{n}^{m}:a_{n+3}^{m})$. The plotted line is: μ+2×σ=0.0025+2×0.0055 bits (top plot) and 0.0022+2×0.0027 bits (bottom plot), this approximates the average amount of “outlier” information we can expect from data that is not related but has similar statistical properties. Estimates of other measures of random information are of similar order.

**Figure 4:**
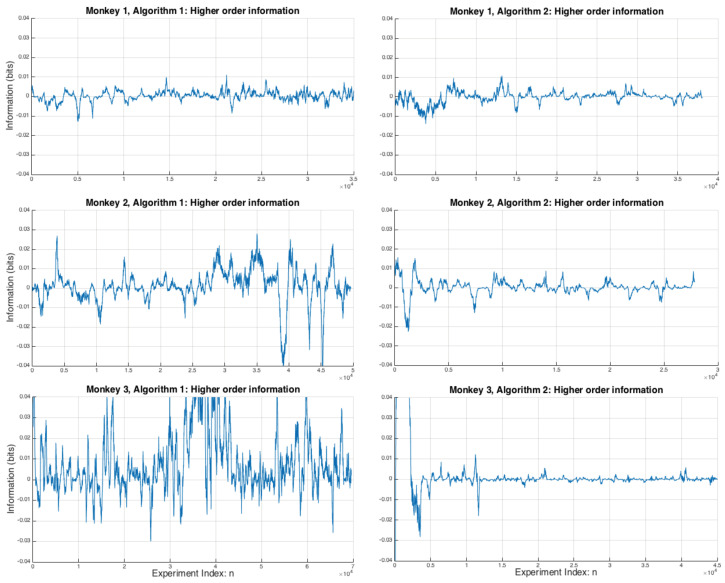
$\delta I(S_{n}:a_{n+1}^{m})$ for all three monkeys across Algorithms 1 and 2 showing the degree of higher order nonlinearities between the elements of $S_{n}$ and $a_{n+1}^{m}$ when choosing $a_{n+1}^{m}$. There is significant variation between the monkeys, but notably Monkey 3 has by far the largest variation under Algorithm 1, and very large transient values of $\delta I(S_{n}:a_{n+1}^{m})$ before settling down to very low degree of nonlinearity under Algorithm 2.

In order to look at other potential sources of information from the previous step we next consider the total information $I(S_{n}:a_{n+1}^{m})$, which is a strict upper bound on the amount of information available from the previous time step, all of the pairwise information sources $I(a_{n}^{m}:a_{n+1}^{m})$, $I(u_{n}:a_{n+1}^{m})$, $I(a_{n}^{c}:a_{n+1}^{m})$ shown in Figure 2 and in more detail in Figure 3, and the difference:

$$\delta I\left(S_{n}:a_{n+1}^{m}\right)=I\left(S_{n}:a_{n+1}^{m}\right)-I\left(a_{n}^{m}:a_{n+1}^{m}\right)-I\left(u_{n}:a_{n+1}^{m}\right)-I\left(a_{n}^{c}:a_{n+1}^{m}\right)$$

shown in Figure 4. It can be seen that for all three monkeys when playing against Algorithm 1 there is a general tendency to increasing values of total information used by the monkeys over the sequence of experiments, this is most pronounced for Monkey 3 where the total amount of information used by the end of the experiments is nearly one bit, the theoretical upper limit for a binary choice. In these experiments the memory, the amount of information used from each monkey’s previous choices, is significantly lower than for the total information and for Monkey 3 it has noticeably decreased over the course of the experiments under Algorithm 1. The patter of total information usage by the Monkeys is reversed for Algorithm 2, showing the Monkeys are using less and less information from the previous time step in forming their next choices, this decrease is most pronounced for Monkey 3.

The largest pairwise contribution to the total information $I(S_{n}:a_{n+1}^{m})$, shown in black in Figure 2, comes from the monkeys following the reward during Algorithm 1, i.e., $I(u_{n}:a_{n+1}^{m})$ shown in red in Figure 2, and this is also true in the first few thousand experiments of Algorithm 2. Neither the computer’s previous choices $a_{n}^{c}$ nor the monkeys’ previous choices $a_{n}^{m}$ have a consistently strong pairwise relationship with $a_{n+1}^{m}$. Note that by the time the experiments finish for Algorithm 1, monkey 3 uses almost one bit of information from its most recent past reward in order to make their next choice.

Once the computer conditions the hypotheses to tests on both past actions and rewards, the extent to which the monkeys extract information from past rewards decreases significantly, and note that there is a redundancy in these conditional probabilities: $p\left(a_{n+1}^{m}|a_{n}^{c}\right)\equiv p\left(a_{n+1}^{m}|\left\{a_{n}^{m},u_{n}\right\}\right)\neq p\left(a_{n+1}^{m}|S_{n}\right)$, a point that was considered in the context of information theory in the previous article [5]. In order to see the effect of this conditioning on the strategies of the monkeys, Figure 3 is an expanded view of the first 12,000 experiments for Monkeys 2 and 3 for Algorithm 2. For both monkeys it can be seen that their are significant strategic variations over time as the pairwise interactions between their next and either their previous choice $I(a_{n}^{m}:a_{n-1}^{m})$ or the reward they previously received $I(a_{n}^{m}:u_{n-1})$.

In Figure 4 $\delta I(S_{n}:a_{n+1}^{m})$ is plotted for all monkeys and both algorithms. This is a measure of the deviation from pairwise independence and acts as a lower bound on the amount of information in the higher order interactions between the variables, see [27,28] for a detailed discussion on the partial information decomposition of the XOR gate, which is formally equivalent to the logic of the matching pennies game [5]. It can be seen hat Monkey 3 (Algorithm 1) has a greater degree of divergence from pairwise independence in its strategies, suggesting that there may be higher order, i.e., more complex, interactions between the variables that are not being picked up by the pairwise information measures. However Monkey 3 also has the lowest $\delta I(S_{n}:a_{n+1}^{m})$ when playing against Algorithm 2 (after an initial transient of the first few thousand games).

Note that in the matching pennies game, and assuming that a strategy is made up of an active memory term and some other contributions, it is possible to decompose the total information into the active memory component and a transfer entropy component in two different ways (using Corollaries 1 and 3 in [5]):

(8) $$\begin{array}{ccc} I(S_{n}:a_{n+1}^{m}) & = & T_{a^{c}\to a^{m}}+I\left(a_{n}^{m}:a_{n+1}^{m}\right) \end{array}$$

(9) 1234567891=Tu→am+I(anm:an+1m)

This shows that we cannot distinguish between the source of the remaining information, it is from either the utility or the computer’s choice but not both, once we have correctly accounted for the memory. However, the results make it quite clear that there is a strong relationship between the reward and the choices the monkey makes, suggesting a possible alternative interpretation of these results.

A second interpretation is to look at the results shown in Figure 2 and conclude that, because the most significant relationship is between the reward from the previous round and the next choice: $I(u_{n}:a_{n+1}^{m})$, then this relationship should be accounted for directly and then any other source of information should be conditional on the reward. This suggests one of these two forms might be the better way to write the total information:

(10) $$\begin{array}{ccc} I(S_{n}:a_{n+1}^{m}) & = & I\left(a_{n}^{c}:a_{n+1}^{m}|u_{n}\right)+I\left(u_{n}:a_{n+1}^{m}\right) \end{array}$$

(11) 01234567891=I(anm:an+1m|un)+I(un:an+1m)

Either of these two formulations would suffice, however, from the results shown above the monkey’s memory term seems to play a slightly more significant (pairwise) role than the computer’s previous choice, but it should be emphasised that the largest single pairwise signal observed in these experiments is from the reward to the monkey’s next choice. We note that because the WSLS strategy is (behaviourally) indistinguishable from the TfT strategy, and because TfT is just a copy of the other player’s previous move measured by $I(a_{n}^{c}:a_{n+1}^{m})$, we might conclude that there is no strong evidence for the WSLS strategy; this will be covered in more detail in the discussion below.

### 3.3. Two-Step Monkey Memory and Conditional Mutual Information

**Figure 5:**
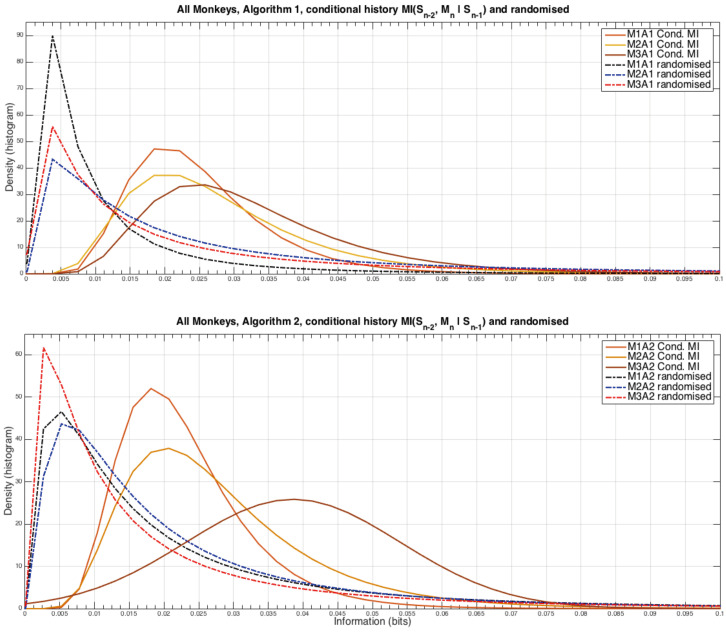
Analysis of the monkeys looking further into their strategic past than just the previous time step. Note that the randomised data peaks at a point at which the original data has almost zero occurrences, strongly suggesting that the observed information measures are statistically different from random chance. However, note that these levels of information are very small compared the amount of information the monkeys make use of from $S_{n}$, as discussed in the other results above.

An important assumption in many theoretical models and simulations of agents playing iterated games is the degree to which the agents make use of information further back in the game history than just the previous step, for example to what extent are an agent’s strategies 1-step Markovian or 2-step Markovian. We tested this point by measuring the mutual information between the second to last time step and the monkeys’ next choice but conditioning out the intermediate behaviour, i.e., $I(S_{n-1}:a_{n+1}^{m}|S_{n})$, the results showed no significant temporal structure as our previous analyses did, and the information measures were significantly smaller as well. With this in mind for clarity we plot the distributions of the information measures rather than the time evolution, the results are shown in Figure 5.

The densities were modelled using a log-normal distribution from the suite of statistical analysis functions in Matlab (R2014b, MathWorks, Natick, Massachusetts, USA). There are distinct and very consistent differences between the randomised (see the caption to Figure 3 for the method) and non-randomised estimates of the amount of information that the monkeys are able to use from two steps into the past interactions. In particular when playing against Algorithm 2, Monkey 3 appears to extract more information than the other monkeys from earlier games. From this result it can be seen that the Markov property that was assumed for the theoretical results in the previous article [5] is violated, but the degree of this violation is relatively mild.

## 4. Discussion

Previous work on this same dataset using conventional parametric statistical analysis has shown that there is statistically significant, but weak, evidence that the WSLS strategy is used by these monkeys [19,29]. However, the WSLS strategy is quite complex, it is equivalent to an XNOR operation in logic gates, and learning an XNOR operation is complex enough to need a multilayer neural network [30], unlike the TfT strategy which is a linear problem and can be solved by a single perception. What these results show is that even in a common game theory task, accounting correctly for all of the contributions to a given behavioural outcome is difficult. In general the decomposition of the total information from $S_{n}$ to $a_{n+1}^{m}$ is complex, but the structure in this experiment is such that some useful simplifications allow us to reduce the complexity to just two information terms that need to calculated, but there are still six ways of writing these two terms (see Equations (6a)–(6f) in [5]), all of which are equally valid decompositions, and so when interpreting these results some assumptions need to be made.

We note that the WSLS strategy is identical with the XNOR (⊕) logic gate: in Equation (6) $Z_{WSLS}^{m}\left(S_{n}\right):u_{n}⊕a_{n}^{m}=a_{n+1}^{m}$ and the TfT strategy: $Z_{TfT}^{m}\left(S_{n}\right):a_{n}^{c}=a_{n+1}^{m}$ are equivalent: $Z_{TfT}^{m}\left(S_{n}\right)\equiv Z_{WSLS}^{m}\left(S_{n}\right)$, as can be seen in the following table [5]:

XNOR logic gate for win-stay, lose-switch strategy of agent *i*
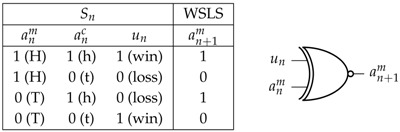


In the case of equal probabilities across the inputs $u_{n}$ and $a_{n}^{m}$ we have $I(u_{n}:a_{n+1}^{m})=0$ bits and $I(a_{n}^{m}:a_{n+1}^{m})=0$ bits but the “synergy” between these variables means that: $I(a_{n}^{m},u_{n}:a_{n+1}^{m})=1$ bit [28] (Section 2.3). In general though for the monkey data presented here, while the pairwise information measure $I(a_{n}^{m}:a_{n+1}^{m})≃0$, it is not even approximately true that $I(u_{n}:a_{n+1}^{m})≃0$ for most of the time under Algorithm 1 as well as for a transient learning period at the beginning of Algorithm 2 for all three monkeys. In particular, if Monkey 3 were to be using this WSLS strategy towards the end of its sessions with Algorithm 1 (Figure 2, bottom left) we should expect to see almost no pairwise relationship between past reward and future choices.

We also note that not only the player’s strategy but also the game itself is a logic gate: the matching pennies game is either an XNOR gate or an XOR gate (depending on which player’s utility is being considered) that relates the variables $a_{n}^{c}$, $a_{n}^{m}$, and $u_{n}$ to one another: $a_{n}^{m}\;⊕\;a_{n}^{c}=u_{n}$. This means there is a redundancy of information in these three variables at time *n* and consequently an ambiguity in the strategy being used by a monkey in making a choice at n+1 based on the previous state-space $S_{n}$. If $I(a_{n}^{c}:a_{n+1}^{m})=1$ bit then we might conclude that a monkey was playing either the TfT strategy or the WSLS strategy, see Theorem 4 of [5]. By symmetry, if $I(u_{n}:a_{n+1}^{m})≃1$ bit (i.e., Figure 2, bottom left) then either the monkey is following the reward from the previous round or its strategy is to compute an XNOR relationship between its previous choice and the computer’s previous choice, but either way it is not computing and XNOR relationship between the utility and its previous choice as the WSLS strategy requires. This can be observed directly in the table above by noting that if there were uniform marginal probabilities for all three variables, then there is zero relationship between the utility $u_{n}$ and the WSLS strategy.

Conventional statistical analysis can make it difficult to estimate on what basis an agent makes its decisions. In earlier work using this data, the probability of the WSLS strategy was found to be statistically significant but not much above chance [19] and that a reinforcement learning model (i.e., reward following) may be a better fit [31], this is consistent with what has been found here, WSLS would have to be a much smaller component of the total information $I(S_{n}:a_{n+1}^{m})$ than the proportion contributed directly by the reward $I(u_{n}:a_{n+1}^{m})$.

Another approach to addressing the statistical inference issue was used in the original studies [19]: take direct recordings of neural activity in the monkeys’ prefrontal cortex and measure the correlations between strategies. The author’s showed that all three elements of the previous times step, previous choice, computer’s choice, and reward, were encoded by the monkeys. The author’s proposed the following simple reinforcement learning model that was consistent with their neural data and other, commonly used, economic models:

(12) $$\begin{array}{ccc} P(R) & = & \frac{\exp(\beta Q(R))}{\exp(\beta Q(R))+\exp(\beta Q(L))} \end{array}$$

in which P(R) is the probability of choosing the right-side option, β is a memory attenuation parameter, and Q(·) is the subjective *value function* associated with either the *L*eft or the *R*ight option. The significance of these recordings is not yet clear: why would the animals encode unused information? These concerns are closely related to the work of the minimal necessary computations that underlie any observed behaviour [32] and the recent work of the subjective internal world of an agent and its objective behaviour in the world [33], these ideas will be explored in future work. These results support the reinforcement model the previous authors proposed while highlighting the theoretical limitations to inferring strategic computations from observing solely behavioural data, contrary to revealed preference theory of economics, as highlighted in the previous study [5].
